# Impact of enteral Ecoimmunonutrition on immunological response, nutritional status and tolerance to treatment in gastrointestinal malignancy patients receiving chemotherapy

**DOI:** 10.1007/s00520-025-10257-7

**Published:** 2025-12-12

**Authors:** Jie Zhi, Bin Wang, Wujie Zhao, Hongyu He, Gang Cheng, Xiaowei Zhang, Bo Feng, Yitao Jia

**Affiliations:** 1 Department of Oncology, Hebei General Hospital, Shijiazhuang, 050051 Hebei Province China; 2 Department of Oncology, North China University of Science and Technology Affiliated Hospital, Tangshan, 063000 Hebei Province China; 3 Department of Nutrition, Hebei General Hospital, Shijiazhuang, 050051 Hebei Province China

**Keywords:** Gastrointestinal malignancies, Enteral immunonutrition, Chemotherapy, Immune modulation, Quality of life

## Abstract

**Purpose:**

Gastrointestinal (GI) malignancies are major contributors to global cancer-related mortality, with many patients experiencing severe nutritional decline and immune suppression due to chemotherapy. Enteral immunonutrition (EIN), which includes immune-modulating nutrients, has shown promise in improving nutrition, reducing chemotherapy-related side effects, and enhancing immune function, but its role in advanced GI cancer patients undergoing chemotherapy is not well-studied.

**Methods:**

This randomized, controlled study involved 28 patients with advanced GI malignancies, assigned to either standard nutrition or EIN for 42 days. Key measures included nutritional status, immune markers, quality of life (QoL), and chemotherapy-related toxicities. The study also used a preclinical mouse model to evaluate EIN's impact on tumor growth and intestinal health during chemotherapy.

**Results:**

Patients in the EIN group demonstrated significantly improved serum albumin levels on day 28 (*P* = 0.03) and a higher CD4 + /CD8 + T-cell ratio on day 42 (*P* < 0.01) compared to controls. EIN supplementation significantly mitigated chemotherapy-induced fatigue and improved QoL scores on days 28 and 42 (*P* < 0.05). Repeated measures analysis revealed a substantial reduction in pro-inflammatory cytokines (IL-1, IL-6) and an increase in the anti-inflammatory cytokine IL-10 in the EIN group (*P* < 0.05). Preclinical findings showed that EIN significantly reduced tumor volume (*P* < 0.05) and preserved the integrity of the intestinal mucosal barrier, evidenced by higher ZO-1 and Occludin expression (*P* < 0.05).

**Conclusion:**

These findings suggest that EIN during chemotherapy enhances nutritional and immune status, reduces inflammation, and improves QoL, warranting further large-scale trials to confirm its benefits in cancer care.

Trial Registration: This trial was registered on the Chinese Clinical Trial Register (ChiCTR2400084224) on 13–05-2024.

**Supplementary Information:**

The online version contains supplementary material available at 10.1007/s00520-025-10257-7.

## Introduction

Gastrointestinal (GI) malignancies are a major cause of cancer-related deaths globally [1]. Many patients are diagnosed at advanced stages, where surgery is not an option [2], leaving systemic chemotherapy as the main treatment [3]. However, chemotherapy often causes severe side effects, including malnutrition, immune suppression, and compromised quality of life (QoL), which can worsen clinical outcomes [4].

Malnutrition affects up to 80% of cancer patients and is particularly concerning in GI cancers due to impaired digestion and nutrient absorption [5]. Malnutrition not only decreases chemotherapy tolerance but also increases morbidity, mortality, and lowers survival rates [4]. Addressing nutritional deficits has become crucial to improving treatment tolerance and outcomes [6].

Enteral immunonutrition (EIN) offers a combination of high-quality nutrients and immune-modulating components, such as ω−3 fatty acids, arginine, and nucleotides. Previous studies have shown that EIN can improve nutrition, reduce infection, and shorten hospital stays in surgical oncology patients [7–9]. However, its role in non-surgical GI cancer patients undergoing chemotherapy remains underexplored, with limited data on its effects on immune modulation, chemotherapy toxicity, and QoL.

In this study, QoL was assessed using the EORTC QLQ-C30, which captures both functioning and symptom domains relevant to chemotherapy in GI malignancies [10, 11]. The key domains of interest included global health/QoL and physical/role functioning, alongside symptom scales—particularly fatigue and appetite loss, as well as pain, nausea/vomiting, and diarrhea. These domains commonly deteriorate during systemic therapy and are mechanistically linked to treatment-related inflammation and altered energy metabolism. Given the immuno-metabolic profile of enteral immunonutrition, we hypothesised that EIN could preferentially attenuate fatigue and appetite loss and support physical functioning during chemotherapy.

EIN may also help reduce systemic inflammation, which is linked to cancer progression and chemotherapy resistance [12]. Pro-inflammatory cytokines like IL-1, IL-6, and TNF-α are often elevated in cancer, contributing to cancer cachexia and reduced treatment efficacy [13]. Anti-inflammatory cytokines like IL-10 may counterbalance these effects, improving clinical outcomes [14]. Understanding EIN’s impact on inflammation during chemotherapy is crucial.

This exploratory randomized study was designed to evaluate whether EIN administered alongside chemotherapy could improve nutritional status and influence secondary outcomes including immune function, chemotherapy-related toxicities, and health-related quality of life (EORTC QLQ-C30), with particular attention to fatigue and appetite loss. EIN provides immune-modulating substrates such as ω−3 polyunsaturated fatty acids, arginine, and nucleotides, which may down-regulate pro-inflammatory signaling and support adaptive immunity during cytotoxic therapy. As chemotherapy in gastrointestinal malignancies is commonly associated with systemic inflammation, mucosal injury, anorexia, and fatigue—symptoms plausibly responsive to immunonutrition—and given that previous studies in surgical oncology populations have demonstrated nutritional and immunological benefits, it was hypothesized that EIN would preserve or improve serum albumin, enhance immune balance (higher CD4 +/CD8 + T-cell ratio, lower IL-1/IL-6, higher IL-10), reduce fatigue, improve QoL, and, in a complementary CT26 murine model, augment oxaliplatin efficacy while maintaining intestinal barrier integrity (ZO-1, Occludin).

## Materials and methods

### Study population

Clinical data were collected from 28 patients with gastrointestinal (GI) malignancies who were admitted to our center for chemotherapy between January and December 2021. This study was designed as an exploratory, single-center pilot trial intended to generate preliminary data on feasibility, safety, and biological response to enteral immunonutrition. Therefore, no formal sample size calculation was performed. The target enrollment of 28 patients reflected the number of eligible participants available within the recruitment window and was consistent with sample sizes commonly used in pilot immunonutrition trials (typically 20–40 patients).

All patients provided informed consent before specimen collection and procedures. The study was approved by our hospital’s Ethics Committee (approval no: 2019/181) and registered at ClinicalTrials.gov (ChiCTR2400084224).

Inclusion criteria were: (1) patients with histologically confirmed esophageal, gastric, or colorectal cancer, unresectable, and scheduled for chemotherapy; (2) locally advanced (stage III) or metastatic (stage IV) disease as per the AJCC staging manual (8th edition); (3) ability to consume food orally; (4) expected survival of at least 3 months; (5) requirement for biweekly or triweekly systemic chemotherapy; (6) age 18–80 years.

Exclusion criteria included: (1) hepatic or renal insufficiency, unmanageable metabolic disorders; (2) active infections; (3) steroid or immunosuppressant use; (4) concurrent malignancies; (5) complete intestinal obstruction; (6) inability to consume food orally; (7) allergy to the investigational product or inability to complete the study.

### Randomization and intervention

Patients were randomly assigned using a random number table to either the control group or the EIN group (n = 14 in each group). The control group received dietary counseling to follow a standard diet, along with general health education. Dietary counseling in both groups followed the Chinese Society for Parenteral and Enteral Nutrition (CSPEN) clinical guidelines, targeting an energy intake of 25–30 kcal/kg/day and 1.0–1.2 g protein/kg/day. The standard hospital diet provided approximately 55–60% of total energy from carbohydrates, 25–30% from fat, and 15–20% from protein. Daily caloric intake was estimated through hospital meal records and patient dietary logs. Minor variations were observed among participants according to appetite and tolerance, but average caloric intake did not significantly differ between groups during the 42-day intervention period.

The EIN group received the same dietary counseling and standard diet, in addition to 237 mL of Ayunutri daily for 42 days, starting on the first day of chemotherapy. The EIN formula used in this study was Ayunutri (TOT BioPharm, Suzhou, Jiangsu Province, China), a whole-nutrition liquid preparation specifically formulated for oncology patients. Each 237 mL jar provided 308 kcal (1.3 kcal/mL). The macronutrient energy distribution was 31% carbohydrates, 45% fats, and 24% proteins. Each jar contained 18.5 g of high-quality protein derived from lactalbumin, casein, and soybean protein. Fat sources included rapeseed oil, safflower seed oil, flaxseed oil, and fish oil, supplying both monounsaturated and polyunsaturated fatty acids, including ω−3 and ω−6 PUFAs. Immune nutrients—arginine and nucleotides—were added to support immunomodulation. Each jar also contained 3.8 g of dietary fiber, composed of oligofructose and resistant dextrin (prebiotic components). Ayunutri is supplied as a ready-to-use liquid suitable for oral or enteral administration. Ayunutri was administered orally (not via nasogastric tube) as a once-daily supplement in addition to the regular diet. Each patient consumed one sealed 237 mL jar per day, equivalent to 308 kcal (1.3 kcal/mL). Intake was supervised by a clinical dietitian, and adherence was verified through daily consumption logs and container checks. No patients required or received nasogastric or enteral tube feeding during the study period.

Before starting the supplementation, patients received detailed education on the correct timing and method of Ayunutri intake. Adherence was monitored through weekly telephone follow-ups conducted by the study dietitian to confirm daily intake and document any side effects or missed doses. Participants were also asked to return empty Ayunutri containers at each hospital visit. Adherence was calculated as the proportion of days with full supplement consumption, which exceeded 90% for all patients.

### Clinical data collection

Baseline patient characteristics were recorded, including name, biological sex, age, hospitalization serial ID, admission/discharge dates, height, weight, body mass index (BMI), comorbidities (hypertension, diabetes), tumor site, stage, and grade.

The primary outcome was change in nutritional status, assessed by serial serum albumin levels measured on days 0, 21, and 42. Secondary outcomes included immune function parameters (CD4 +/CD8 + T-cell ratio, cytokine profiles), chemotherapy-related adverse effects (graded by NCI-CTCAE), and patient-reported quality of life (EORTC QLQ-C30).

### Nutritional risk and status assessments

Nutritional risk was assessed using the Nutrition Risk Screening 2002 (NRS-2002), with a score of ≥ 3 indicating nutritional risk. Nutritional status was evaluated using the Scored Patient-Generated Subjective Global Assessment (PG-SGA©), with scores categorized as good nutrition (0–1), probable malnutrition (2–3), moderate malnutrition (4–8), and severe malnutrition (≥ 9).

### Quality of life assessment

The European Organization for Research and Treatment of Cancer Quality of Life Questionnaire (EORTC QLQ-C30, version 3) was used to assess quality of life (QoL), where higher scores indicate better QoL.

### Chemotherapy toxicity assessment

Chemotherapy-related adverse events, including myelosuppression, gastrointestinal reactions, hepatic impairment, diarrhea, and fatigue, were graded using the National Cancer Institute Common Terminology Criteria for Adverse Events (NCI-CTCAE). These assessments were conducted at fixed time points (days 0, 7, 21, 28, and 42) regardless of each patient’s specific chemotherapy infusion schedule. As a result, some assessments may have occurred before or after chemotherapy cycles, which could influence the severity and timing of reported toxicities.

### Immune response analysis (Flow Cytometry)

Peripheral venous blood (5 mL) was collected on day 0 (pre-chemotherapy), day 21, and day 42 after an overnight fast. Samples were anticoagulated with EDTA. The numbers of CD4 + and CD8 + T cells and the CD4 +/CD8 + T cells ratio were determined using a FACS Aria™ II flow cytometer (BD Biosciences, USA). Antibodies used included CD45-FITC, CD3-PC5, CD4-RD1, and CD8-ECD (all Abcam, USA). Data were analyzed using FlowJo v10 software.

### Cytokine quantification (ELISA)

Serum levels of IL-1, IL-6, IL-8, IL-10, and TNF-α were measured at three time points (day 0, 21, and 42) using ELISA kits (ALCO, Cambridge, UK), following centrifugation of blood samples at 3500 rpm for 10 min. Mouse blood samples were obtained post-intervention for similar cytokine analysis, using an ELISA kit (ABclone, Wuhan, China).

### Preclinical mouse model

The animal experiments aimed to test the hypothesis that EIN could potentiate the antitumor effects of oxaliplatin (L-OHP) and protect intestinal mucosal integrity during chemotherapy. Twenty-four male BALB/c mice (6 weeks old, 20–25 g) were purchased from Hebei Medical University (quality certificate No. 2010069) and housed under a 12-h light/dark cycle with ad libitum food and water. CT26 mouse-derived colon cancer cells (2 × 10^5^) were subcutaneously injected into the right forelimb to establish xenografts. The full details of the animal experiments can be found in Supplemental File.

All experimental procedures were approved by the Institutional Animal Care and Use Committee of Hebei General Hospital (approval no: 2020-DW-168) and complied with the National Guidelines for Laboratory Animal Welfare (GB/T 35892–2018) and the ARRIVE 2.0 guidelines.

### Animal grouping and interventions

Mice were randomly divided into three groups (n = 8 per group): control, L-OHP (oxaliplatin), and EIN + L-OHP. The control group received saline (0.2 mL daily). The L-OHP group received intraperitoneal oxaliplatin (5 mg/kg every two days) and daily saline. The EIN (Ayunutri, TOT BioPharm, Suzhou, China) preparation is supplied in liquid form and was administered directly by oral gavage at a volume of 0.2 mL once daily. This corresponds to approximately 10 mL/kg body weight for BALB/c mice weighing 20–25 g, which is the standard and well-tolerated gavage volume recommended for nutritional studies. The dose was selected based on published murine immunonutrition protocols and pilot tolerance observations performed before the study. Tumor volumes were measured bi-daily using the formula V = 1/2 A × B^2^, and overall health, including body weight, fur quality, and activity, was monitored.

### Histological and immunohistochemical analyses

Mouse colon tissue was paraffin-embedded, sectioned (5 μm), and stained with hematoxylin and eosin (H&E) for histopathological assessment. ZO-1 and Occludin expression were analyzed via immunohistochemistry using rabbit anti-mouse antibodies (GeneTex, Taiwan). The average optical density (OD) was calculated using Image-Pro Plus 6.0.

### Statistical analysis

All data were analyzed using SPSS 21.0 software. Continuous variables were expressed as mean ± standard deviation (¯x ± SD), and categorical data as frequencies. Intergroup comparisons were conducted using independent sample t-tests, while repeated measures ANOVA was used for longitudinal data. Chi-square tests were applied to categorical variables. The normality of continuous data was evaluated using the Shapiro–Wilk test before applying parametric analyses. Normally distributed data were analyzed using independent-sample t-tests and repeated-measures ANOVA, while non-normally distributed variables were assessed using Mann–Whitney U or Friedman tests, as appropriate. A *P*-value < 0.05 was considered statistically significant.

## Results

### Baseline characteristics

A total of 28 patients with GI malignancies were enrolled between January and December 2021, including 19 males (67.86%) and 9 females (32.14%) with a median age of 64.5 years (range: 38–79 years). As shown in Table S1, there were no significant differences between the EIN and control groups regarding diabetes mellitus, hypertension, biological sex, age, BMI, tumor site, stage, or grade.

### Impact of EIN on nutritional risk and status

The EIN group maintained higher albumin levels than the control group, with a significant difference noted on day 28 (38.24 ± 3.12 g/L *vs.* 35.56 ± 2.91 g/L; *P* = 0.03), but not at other time points (all *P* > 0.05). No significant changes in albumin levels over time were observed within either group (*P* > 0.05). Both nutritional status (PG-SGA) and nutritional risk (NRS-2002) did not differ significantly between the groups (Table S2).

### Immune effects of EIN on CD4 +/CD8 + T cell ratios and T cell levels

By day 42, the EIN group exhibited significantly fewer CD8 + T cells (25.03 ± 12.27 *vs.* 34.13 ± 0.17; *P* = 0.03), and a higher CD4 + T cell count (41.90 ± 7.75 *vs.* 35.53 ± 8.83;* P* = 0.04) with an increased CD4 +/CD8 + T cell ratio (2.09 ± 0.28 *vs. *1.32 ± 0.17; *P* < 0.01). No significant differences in immune parameters were observed between the groups at day 21, nor were there substantial changes over time within the EIN group (**Table S3**).

### Effect of EIN on chemotherapy toxicity and quality of life

Fatigue worsened significantly in the control group (*P* < 0.05), but remained stable in the EIN group (*P* > 0.05). Fatigue improvement in the EIN group was notably higher on days 7, 28, and 42 (7/14 (50%) *vs. *1/14 (7.1%), 9/14 (64.3%) *vs.* 2/14 (14.3%), and 9/14 (64.3%) *vs.* 1/14 (7.1%), respectively; all *P* < 0.05). There were no significant differences in chemotherapy-related toxicities between the groups (*P* > 0.05). QoL improved significantly in the EIN group over time (F = 2.941, *P *= 0.03), with better scores on days 28 and 42 compared to controls (*P* = 0.01; *P* < 0.01) (Table 1). Chemotherapy efficacy was not significantly enhanced by EIN (*P* = 0.33). Information regarding the chemotherapeutic side effects in both groups are summarized in Table 2. Statistically significant differences were noted regarding fatigue between both groups at 7, 21, and 28 days (*P* < 0.05).

**Table 1:** The QLQ-C30 quality of life score in patients between the EIN group and the control group

Group	n	Day 0	Day 7	Day 21	Day 28	Day 42
EIN group	14	65.57 (3.50)	62.93 (3.27)	67.14 (2.94)	71.28 (2.71)^a^	72.21 (2.57)^a^
Control group	14	65.00 (2.30)	62.36 (2.08)	61.21 (2.36)	61.71 (2.46)	62.29 (2.07)

^a^statistically significant difference with the control group (*P* < 0.05). EIN: Enteral immunonutrition

**Table 2:** The incidence of chemotherapeutic side effects in patients observed in EIN group and the control group

Group	Number	Myelosuppression	Gastrointestinal reaction	Hepatic injury	Diarrhea	Fatigue
EIN group	14					
Day 0		5 (35.7)	5 (35.7)	3 (21.4)	0 (0)	3 (21.4)
Day 7		6 (42.9)	8 (57.1)	1 (7.1)	1 (7.1)	1 (7.1)^b^
Day 21		9 (64.3)	8 (57.1)	3 (21.4)	1 (7.1)	2 (14.3)
Day 28		8 (57.1)	7 (50)	2 (14.3)	1 (7.1)	2 (14.3)^b^
Day 42		7 (50)	7 (50)	2 (14.3)	0 (0)	1 (7.1)^b^
Control group	14					
Day 0		2 (14.3)	5 (35.7)	0 (0)	0 (0)	0 (0)
Day 7		6 (42.9)	10 (71.4)	2 (7.1)	1 (7.1)	7 (50)^a^
Day 21		6 (42.9)	10 (71.4)	4 (35.7)	1 (7.1)	7 (50)^a^
Day 28		6 (42.9)	11 (78.6)	4 (35.7)	1 (7.1)	9 (64.3)^a^
Day 42		6 (42.9)	10 (71.4)	5 (35.7)	1 (7.1)	9 (64.3)^a^

^a^ statistically significant difference was noted within the same group before treatment (*P* < 0.05); ^b^ statistically significant difference with the control group (*P* < 0.05). Data are presented as frequency (percentage). EIN:Enteral immunonutrition

### Effects of EIN on serum inflammatory cytokines

**Fig. 1:**
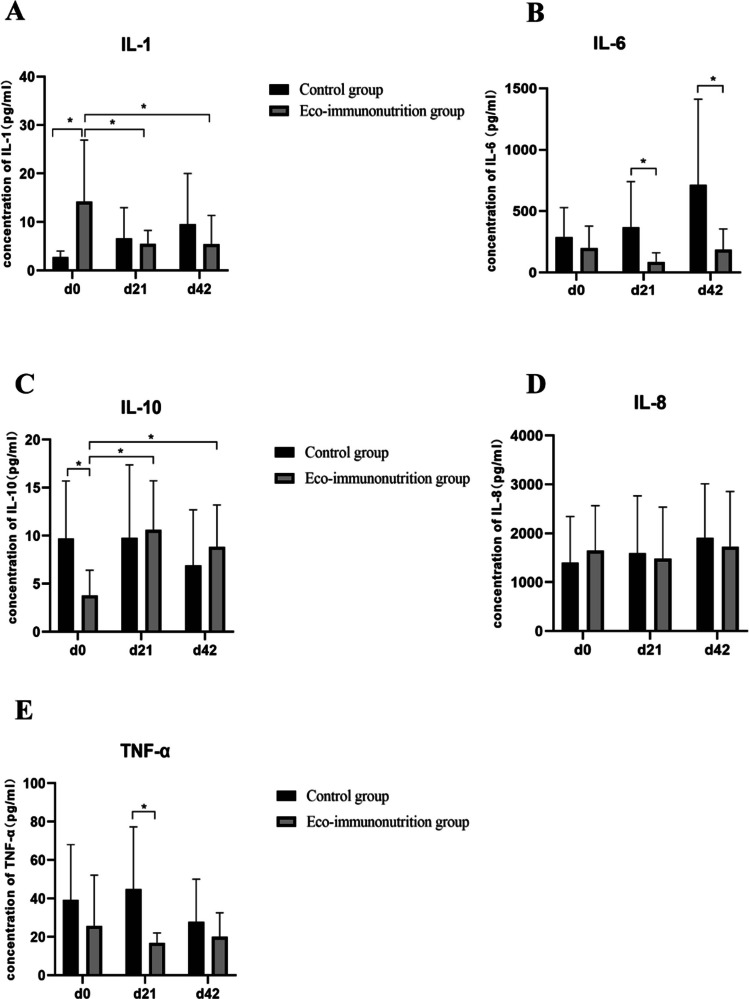
Levels of inflammatory factors in the serum of patients in each group (**A**—IL-1; **B**—IL-6; **C**—IL-10; **D**—IL-8; **E**—TNF-α) (*P < 0.05)

At baseline, the EIN group had higher IL-1 (*P* < 0.01) and lower IL-10 levels (*P* = 0.01) than controls (Fig. 1). Over time, EIN reduced IL-1 and increased IL-10 levels (*P* < 0.05), though specific time point differences were not significant (*P *> 0.05). IL-6 levels were lower in the EIN group on days 21 and 42 (*P* = 0.02), while TNF-α decreased on day 21 (*P* = 0.04). IL-8 levels remained unchanged in both groups.

### Effects of EIN preparations on chemotherapeutic efficacy in colon cancer-bearing mice

#### General conditions of tumor-bearing mice

**Fig. 2:**
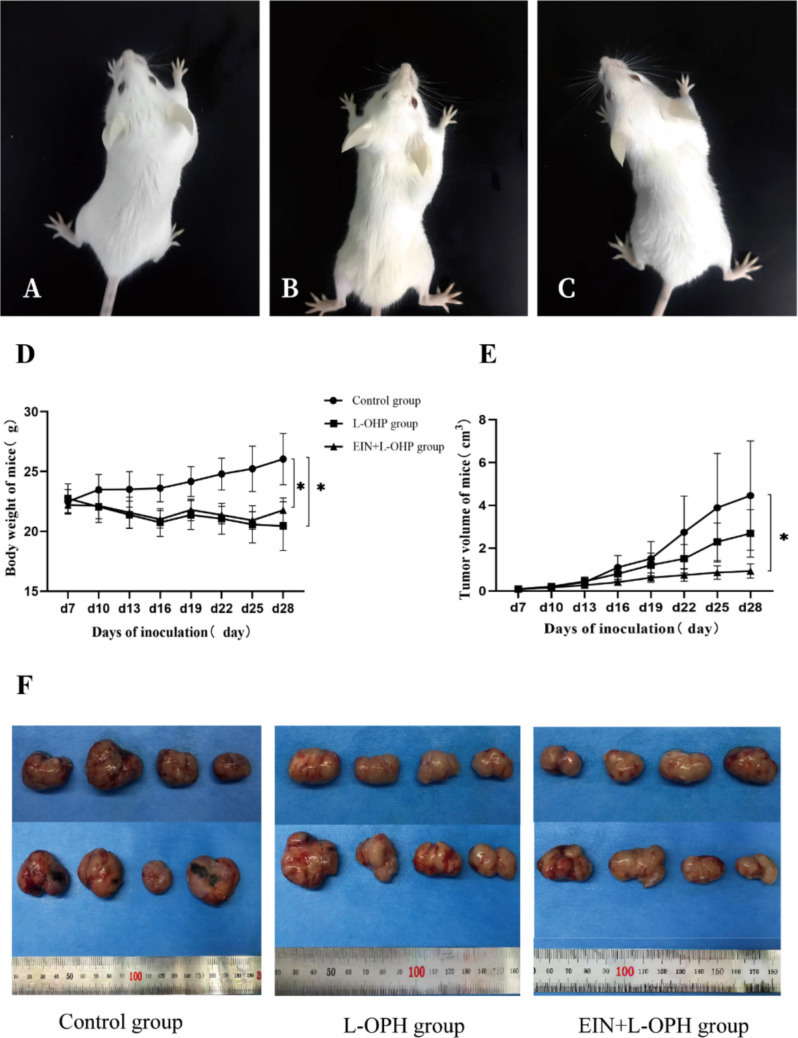
General conditions of mice in each group (**A**—Control group; **B**—L-OHP group; **C**—EIN + L-OHP group); Changes in body weight of mice in each group (**D**); Tumor growth curves of mice in each group(**E**); Tumor tissue from each group of mice (**F**)

No mortality was observed. Mice in the control group were active but overweight with dull fur, while L-OHP group mice appeared depressed and thin with rough fur. EIN + L-OHP mice maintained better physical condition and had glossy fur (Fig. 2A-C).

#### Changes in body weight

Body weight increased in the control group but decreased in both L-OHP and EIN + L-OHP groups. The control group’s final body weight was significantly higher than the L-OHP and EIN + L-OHP groups (*P* < 0.05), though the difference between L-OHP and EIN + L-OHP was not statistically significant (*P* = 0.12) (Fig. 2D).

#### Tumor growth

Tumor volume decreased significantly in the EIN + L-OHP group from day 16 (*P* < 0.05), but no significant difference in tumor volume was seen between the L-OHP and control groups (*P* > 0.05) (Fig. 2E-F).

#### Changes in the intestinal mucosal barrier

**Fig. 3:**
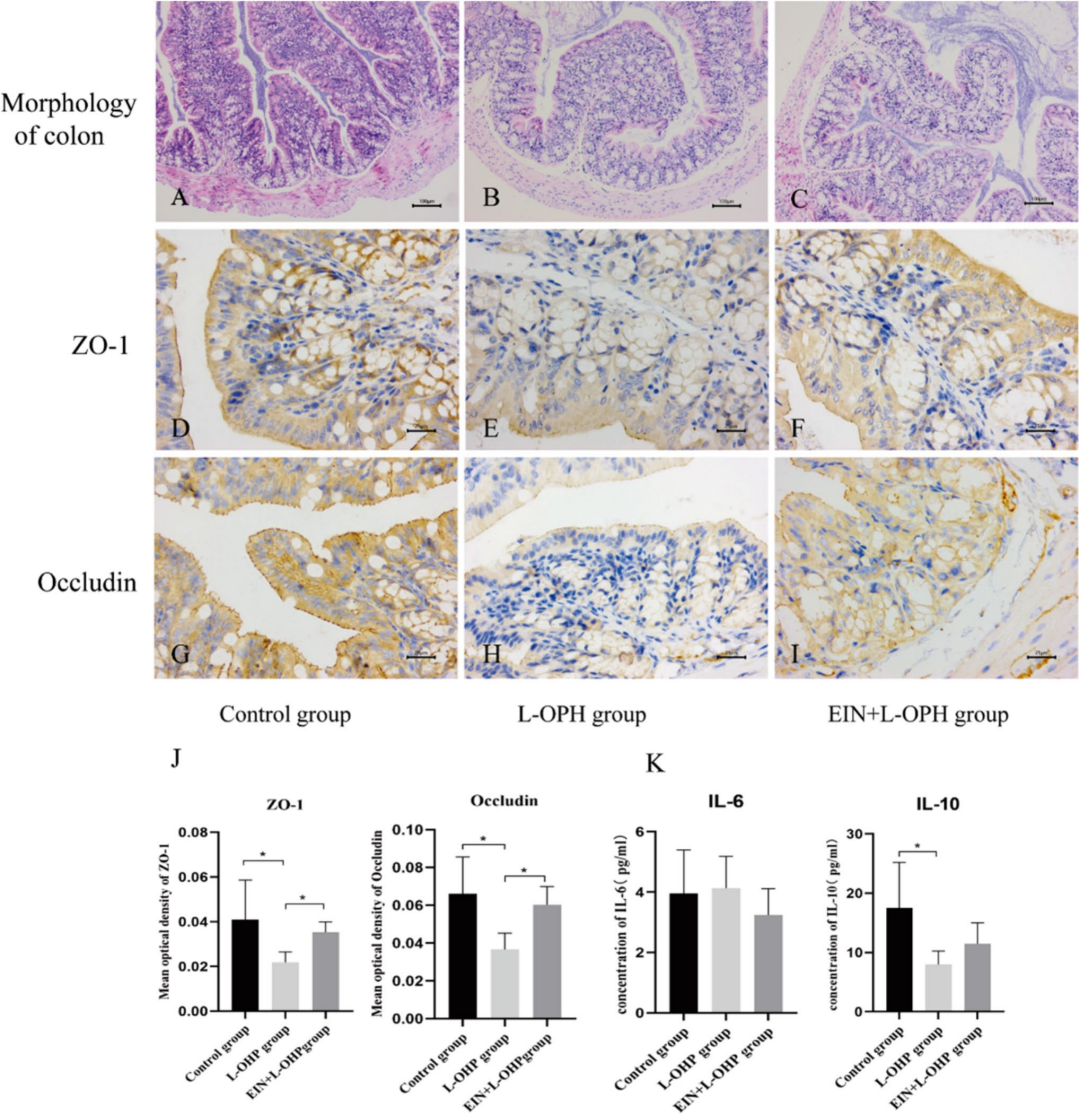
The morphological changes of colon in each group of mice (HE, 100 ×) (**A**—Control group; **B**—L-OHP group; **C**—EIN + L-OHP group) bar = 100 μm;The expression of ZO-1 protein and Occludin protein in colon tissue of each group (IHC, SP method, 400 ×) (**D**, G—Control group; **E**, H—L-OHP group; **F**, I—EIN + L-OHP group) bar = 25 μm; The expression of ZO-1 and Occludin protein in each group (*P < 0.05) (**J**); Levels of inflammatory factors in serum of each group in mice (*P < 0.05) (K)

Histopathology showed intact epithelial cells in the control group. The L-OHP group had detached epithelial cells and malformed villi, while the EIN + L-OHP group displayed intact mucosa and fewer inflammatory cells (Fig. 3A-C). ZO-1 and Occludin protein expression decreased significantly in the L-OHP group (*P* = 0.04, *P* = 0.03) but remained stable in the EIN + L-OHP group (*P* = 0.57, *P* = 0.16), with significantly higher levels than the L-OHP group (*P* = 0.04, *P* = 0.02) (Fig. 3J).

#### Changes in serum inflammatory factor levels

Post-chemotherapy, IL-10 levels significantly dropped in the L-OHP group (*P* < 0.01) but remained unchanged in the EIN + L-OHP group (*P* = 0.24). While EIN appeared to increase IL-10, the difference between L-OHP and EIN + L-OHP groups was not significant (*P* = 0.33). Similarly, IL-6 levels did not significantly change in either group, though EIN + L-OHP showed a modest decrease compared to L-OHP, without reaching significance (*P* = 0.56) (Fig. 3K).

## Discussion

This study presents novel insights into the effects of EIN preparations on the nutritional status, immune response, chemotherapy tolerance, and inflammatory cytokine levels in patients with GI malignancies. Additionally, the investigation into the chemotherapeutic efficacy of EIN in a colon cancer-bearing mouse model provides valuable preclinical evidence for the potential synergistic effects of EIN when combined with chemotherapy. Taken together, our findings suggest that EIN supplementation may help alleviate chemotherapy-related fatigue and improve overall quality of life, with trends toward favorable immune modulation. These preliminary results warrant confirmation in larger, adequately powered trials before firm conclusions can be drawn regarding broader clinical benefits.

### Nutritional benefits of EIN

Our results demonstrate that EIN supplementation maintained higher serum albumin levels in patients with GI malignancies compared to the control group, particularly on day 28. This finding is consistent with previous research suggesting that immunonutrition can improve protein metabolism and stabilize nutritional markers in cancer patients undergoing treatment [15, 16]. Although the overall improvement in albumin levels was not statistically significant at earlier or later time points, the trend suggests that EIN may contribute to maintaining better nutritional status over time, particularly in the context of prolonged chemotherapy. The absence of significant changes in the PG-SGA and NRS-2002 scores between groups may reflect the relatively short follow-up period or the complexity of assessing nutrition in cancer patients receiving intensive therapy.

### Immune modulation and T cell response

One of the most striking findings of this study was the modulation of immune markers, specifically the CD4 +/CD8 + T cell ratio and the absolute levels of CD4 + and CD8 + T cells. On day 42, the EIN group exhibited a significantly higher CD4 +/CD8 + T cell ratio compared to the control group, driven by an increase in CD4 + T cells and a concomitant decrease in CD8 + T cells. This is particularly relevant given the well-established role of the CD4 +/CD8 + T cell ratio in predicting immune competence and outcomes in cancer patients [17–19]. The elevated CD4 + T cell levels in the EIN group suggest a potential enhancement of immune surveillance and a more favorable immunological environment, which could contribute to better patient outcomes. Notably, the significant reduction in CD8 + T cell levels in the EIN group may indicate a decreased cytotoxic T cell response, though this reduction was not associated with any clinically significant immunosuppression in our cohort. These findings align with prior studies demonstrating the immunomodulatory properties of immunonutrition in oncology settings [20, 21].

### Chemotherapy tolerance and quality of life

Chemotherapy-induced fatigue is a well-recognized challenge in oncology, with a profound impact on patients’ quality of life. Our study found that EIN supplementation significantly mitigated fatigue in patients receiving chemotherapy, with substantial improvements observed on days 7, 28, and 42. In contrast, the control group experienced a progressive worsening of fatigue throughout the treatment period. These results suggest that EIN may play a role in alleviating one of the most debilitating side effects of chemotherapy, potentially by supporting energy metabolism and reducing inflammation [8, 22]. Importantly, while EIN did not significantly impact other chemotherapy-related toxicities, such as myelosuppression or gastrointestinal symptoms, the marked improvement in QoL in the EIN group, particularly at later time points, underscores the potential of EIN to enhance overall well-being during chemotherapy. The repeated measures analysis revealed a sustained improvement in QoL scores over time, emphasizing the cumulative benefits of EIN supplementation.

### Anti-inflammatory effects of EIN

Inflammatory cytokines play a pivotal role in cancer progression and the response to treatment. Our study showed that EIN supplementation was associated with a significant reduction in IL-1 and IL-6 levels, both key pro-inflammatory cytokines involved in tumor-promoting inflammation. The decrease in IL-1 and IL-6 was accompanied by an increase in IL-10, an anti-inflammatory cytokine, further indicating that EIN may help to restore immune homeostasis in patients with GI malignancies. These findings are in line with previous reports that suggest the anti-inflammatory effects of immunonutrition can mitigate chemotherapy-induced inflammation and enhance the therapeutic response [16, 23, 24]. Although the changes in TNF-α and IL-8 levels were not statistically significant, the trend towards lower TNF-α levels in the EIN group warrants further investigation in larger studies.

### Preclinical findings in colon cancer-bearing mice

The preclinical data from our mouse model of colon cancer further support the clinical findings of this study. EIN supplementation in combination with L-OHP chemotherapy improved the general health and appearance of tumor-bearing mice, which exhibited less weight loss and better coat condition compared to the L-OHP-only group. Importantly, EIN appeared to enhance the chemotherapeutic efficacy of L-OHP, as evidenced by the significant reduction in tumor volume from day 16 onwards. This synergistic effect may be attributed to the improved immune response and anti-inflammatory properties of EIN, which could augment the host’s ability to respond to chemotherapy [23, 25]. The preservation of the intestinal mucosal barrier in the EIN + L-OHP group, as indicated by higher levels of ZO-1 and Occludin, also suggests that EIN may help protect against chemotherapy-induced gut toxicity, a major complication that can limit treatment intensity in cancer patients [26, 27].

### Study limitations

Several limitations must be acknowledged. The small sample size and short follow-up may have limited the detection of significant differences in outcomes like nutritional risk and chemotherapy toxicities. Larger studies with longer follow-ups are needed to confirm these findings and assess EIN’s long-term benefits. Further research is also required to clarify the mechanisms through which EIN modulates the immune response and reduces inflammation, especially in relation to tumor biology and chemotherapy resistance. Given the small sample size, the statistical power to detect more subtle differences was limited. As such, while the findings are suggestive of potential benefits of EIN, they must be interpreted cautiously and considered hypothesis-generating rather than definitive. Larger, multi-center trials are essential to validate these observations and further investigate the mechanisms involved.

Another limitation is the potential variability in dietary intake within and between groups. Although energy and macronutrient targets were standardized and monitored, individual appetite and tolerance differences could have introduced uncontrolled variation, which may partially confound the nutritional outcomes observed.

Furthermore, the restricted use of patient-reported outcome measures. Although CTCAE grading provided standardized toxicity assessment, it lacks the sensitivity to capture specific chemotherapy-related symptoms such as nausea, appetite loss, or fatigue severity. Future studies should integrate comprehensive PROMs (e.g., EORTC QLQ-C30 symptom scales or disease-specific modules) to better reflect patient experience and treatment tolerance.

A notable limitation of our study is the reliance on serum albumin as a nutritional marker. Albumin is an acute-phase reactant influenced by systemic inflammation and treatment effects rather than a direct reflection of nutritional status [28]. The observed difference in albumin levels at day 28 may be attributed to chemotherapy cycles or albumin's half-life, rather than a sustained nutritional benefit of EIN. This is further supported by the absence of significant differences in PG-SGA and NRS-2002 scores between groups. Future studies should incorporate more robust nutritional assessment tools to confirm EIN’s role in maintaining nutritional status.

Another limitation of our preclinical model is the lack of body composition analysis to distinguish between tumor burden, adipose tissue loss, or potential edema as contributors to weight changes. While weight loss in the OHP-treated groups aligns with expected chemotherapy-induced cachexia, future studies should incorporate methods such as dual-energy X-ray absorptiometry (DEXA) or bioelectrical impedance analysis to better characterize the nature of weight fluctuations.

Furthermore, our preclinical model is the exclusive use of male mice, which does not account for potential sex-based biological differences in immune response, chemotherapy tolerance, and tumor progression. Future studies should incorporate both male and female mice to assess sex-specific effects of EIN and chemotherapy. Additionally, our study utilized a subcutaneous xenograft model rather than an orthotopic colorectal cancer model, which may limit the translatability of our findings to human disease. Orthotopic models better mimic the tumor microenvironment, including interactions with the intestinal epithelium and local immune system. Future research should explore EIN’s effects in models that better represent the complexity of colorectal cancer biology.

In conclusion, our findings suggest that EIN may help improve nutritional status, modulate immune response, alleviate fatigue, and enhance QoL in patients with gastrointestinal malignancies undergoing chemotherapy. Preclinical results further indicate a potential synergistic effect with chemotherapy. However, given the exploratory design and small sample size, these observations should be regarded as preliminary and hypothesis-generating. Larger, multicenter randomized controlled trials are required to confirm the efficacy and determine whether EIN can be incorporated into standard supportive care for cancer patients.

## Supplementary Information

Below is the link to the electronic supplementary material.

Supplementary file1 (DOCX 14 KB)

Supplementary file2 (DOCX 21 KB)

## Data Availability

The datasets generated during and/or analyzed during the current study are available from the corresponding author on reasonable request due to privacy.
