# Supplementary material for: Impact of enteral Ecoimmunonutrition on immunological response, nutritional status and tolerance to treatment in gastrointestinal malignancy patients receiving chemotherapy
Source: Support Care Cancer. 2025 Dec 12;34(1):29. doi: 10.1007/s00520-025-10257-7 (PMC12701029; doi:10.1007/s00520-025-10257-7)
Supplement: Supplementary file 1 — Supplementary file1 (DOCX 14 KB) [file 520_2025_10257_MOESM1_ESM.docx]

**Supplemental File**

**Cell lines and reagents**

The mouse-derived colon cancer cell line CT26 was acquired from Procell, located in Wuhan, China. RPMI 1640 medium and fetal bovine serum (FBS) were acquired from Gibco.

**Establishment of a CT26 colon cancer mouse model**

A total of twenty-four SPF-grade male BALB/c mice, aged 6 weeks and weighing approximately 20g-25g, were acquired from Hebei Medical University (quality certificate No. 2010069). The mice were kept in the Clinical Research Center of Hebei Provincial People's Hospital under a 12-hour light/dark cycle, with unrestricted access to food and water. After one week of adaptation, a total of 2×105 CT26 cells were injected just beneath the skin on the back of the right forelimb of each mouse to establish a xenograft tumor model. The date of inoculation was documented as d1.

**Grouping and interventions of experimental animals**

Twenty-four BALB/c mice with tumors were randomly assigned to three groups: the control group, the oxaliplatin monotherapy group (L-OHP group), and the EIN group (EIN+L-OHP group), with eight animals in each group. In the control group, once the tumors reached a size of approximately 50 mm3 -100 mm3, gastric gavage with saline (0.2 mL) was administered daily. The L-OHP group received an equivalent amount of saline as the control group, which was administered once daily. Additionally, they were given an intraperitoneal injection of oxaliplatin at a dosage of 5 mg/kg, once every two days. The mice in the EIN+L-OHP group were administered EIN preparations through gastric gavage at a volume of 0.2 mL once daily. Additionally, they received oxaliplatin through intraperitoneal injection at a dosage of 5 mg/kg once every two days. Each of the therapies had a duration of 3 weeks.

Following subcutaneous implantation, the tumors' longest diameter (A) and shortest diameter (B) were measured every 4 days using a vernier caliper. The tumor volume (V) was then determined using the formula V=1/2 A×B2 (32). Upon reaching a tumor size ranging from 50 mm3-100 mm3, the administration of the drug/placebo commenced, and the average tumor volume for each group was computed. The tumor development curve was plotted as appropriate. The mice in these three groups received a 21-day intervention. The mice were weighed bi-daily following the administration of the drug/placebo, and their overall conditions, including the quality of their fur, mental state, and physical activity, were also monitored.

**Hematoxylin and Eosin (H&E) staining**

Colon tissue from mice was obtained, fixed in paraffin, and cut into slices that were 5-micrometer- thick slices. Following the removal of wax and dehydration, the tissues were subsequently stained with hematoxylin and eosin, respectively. Afterwards, the specimens underwent dehydration using a gradient of ethanol concentrations ranging from 75% to 100%. They were then cleaned in a xylene combination and ultimately mounted in neutral resin.

**Immunohistochemical analysis**

Mouse colon tissue specimens were treated with the appropriate antibodies, namely rabbit anti-mouse occludin polyclonal antibody (1:500) (GeneTex, Taiwan Province, China) and rabbit anti-mouse ZO-1 polyclonal antibody (1:500) (GeneTex), following dewaxing, hydration, antigen repair, and incubation with 3% H2O2 for 10 minutes, respectively. For microscopy, the mixture was subsequently stained with DAB. A total of three high-magnification fields were selected at random for image acquisition during the microscopic observation. Analysis of each field was conducted utilizing the Image-Pro Plus software, version 6.0. Finally, the position of target protein expression was determined, and positive outcomes were denoted by a brown hue. The average optical density (OD) was subsequently computed using the ratio of the cumulative optical density in the image to the area of the region of interest.
