# Supplementary material for: Impact of enteral Ecoimmunonutrition on immunological response, nutritional status and tolerance to treatment in gastrointestinal malignancy patients receiving chemotherapy
Source: Support Care Cancer. 2025 Dec 12;34(1):29. doi: 10.1007/s00520-025-10257-7 (PMC12701029; doi:10.1007/s00520-025-10257-7)
Supplement: Supplementary file 2 — Supplementary file2 (DOCX 21 KB) [file 520_2025_10257_MOESM2_ESM.docx]

**Study Tables**

**Table S1.** Comparison of overall patient conditions between the EIN group and the control group

| Variable | EIN group (%) | Control group (%) | χ^2^value | P value |
| --- | --- | --- | --- | --- |
| Gender; N (%) |  |  | 4.094 | 0.103 ^a^ |
| Male | 12 (85.7) | 7 (50) |  |  |
| Female | 2 (14.3) | 7 (50) |  |  |
| Age (year); M (SD) |  |  | 1.474 | 0.420 ^a^ |
| <60 years | 6 (42.9) | 3 (21.4) |  |  |
| ≥60 years | 8 (57.1) | 11 (78.6) |  |  |
| BMI (kg/m^2^); M (SD) |  |  | 1.037 | 1.00^a^ |
| <18.5 | 0 (0%) | 1 (7.1) |  |  |
| ≥18.5 | 14 (100) | 13 (92.9) |  |  |
| Tumor location; N (%) |  |  | 6.093 | 0.05^a^ |
| Esophageal cancer | 4 (28.6) | 3 (21.4) |  |  |
| Gastric cancer | 6 (42.8) | 1 (7.2) |  |  |
| Colorectal cancer | 4 (28.6) | 10 (71.4) |  |  |
| TNM stage; N (%) |  |  | 2.19 | 0.330 ^a^ |
| III | 1 (7.1) | 4 (28.6) |  |  |
| IV | 13 (92.9) | 10 (71.4) |  |  |
| Tumor grade (Differentiation); N (%) | |  | 0 | 1.00^a^ |
| Moderate | 6(42.9) | 6(42.9) |  |  |
| Poor | 8(57.1) | 8(57.1) |  |  |
| [Diabetes; N (%)](javascript:;) |  |  | 0 | 1.00^a^ |
| Yes | 2 (14.3) | 2 (14.3) |  |  |
| No | 12 (85.7) | 12 (85.7) |  |  |
| [Hypertension; N (%)](javascript:;) |  |  | 0 | 1.00^a^ |
| Yes | 5 (35.7) | 5 (35.7) |  |  |
| No | 9 (64.3) | 9 (64.3) |  |  |

^a^ P-values calculated by Fisher’s test between the EIN group and control group.

**Table S2.** The albumin level in patients between the EIN group and the control group（$\bar{x}$± s）

| **Group** | n | Day 0 | Day 7 | Day 21 | Day 28 | Day 42 |
| --- | --- | --- | --- | --- | --- | --- |
| **EIN Group;** M (SD) | 14 | 39.57 (3.54) | 37.69 (2.92) | 37.84 (3.61) | 38.24 (3.12)^a^ | 37.01 (3.53) |
| **Control Group;** M (SD) | 14 | 37.79 (4.88) | 37.24 (3.46) | 37.30 (5.28) | 35.56 (2.91) | 36.09 (4.49) |

^a^ statistically significant difference with the control group (p<0.05) EIN: Enteral immunonutrition; M: mean; SD: standard deviation.

**Table S3.** The CD8+T, CD4+T cell and CD4+/CD8+ ratio in patients between the EIN group and the control group

| Group | Number | CD8+T | CD4+T | CD4+/CD8+ ratio |
| --- | --- | --- | --- | --- |
| EIN group; M (SD) | 14 |  |  |  |
| Day 0 |  | 29.31 (13.26) | 40.13 (7.93) | 1.71 (0.24) |
| Day 21 |  | 29.52 (13.25) | 39.07 (8.67) | 1.65 (0.23) |
| Day 42 |  | 25.03 (12.27)^a,b^ | 41.90 (7.75)^b^ | 2.09 (0.28)^b^ |
| Control group; M (SD) | 14 |  |  |  |
| Day 0 |  | 31.21 (0.20) | 36.20 (12.2) | 1.37 (0.20) |
| Day 21 |  | 31.16 (0.17) | 37.86 (12.9) | 1.34 (0.17) |
| Day 42 |  | 34.13 (0.17) | 35.53 (8.83) | 1.32 (0.17) |

^a^ statistically significant difference was noted within the same group before treatment (p<0.05); ^b^ statistically significant difference with the control group (p<0.05). EIN: Enteral immunonutrition; M: mean; SD: standard deviation.
